# First person – Khatija Nishat

**DOI:** 10.1242/bio.062269

**Published:** 2025-10-16

## Abstract

First Person is a series of interviews with the first authors of a selection of papers published in Biology Open, helping researchers promote themselves alongside their papers. Khatija Nishat is first author on ‘ IFT139 regulates Hedgehog signaling and cilia structure through ciliary protein localization’, published in BiO. Khatija is a post-undergraduate in the lab of Dr Yulu Cherry Liu at Hood College, Frederick, USA, investigating how primary cilia and intraflagellar transport proteins regulate cell signalling and development, with implications for ciliopathy-related diseases.


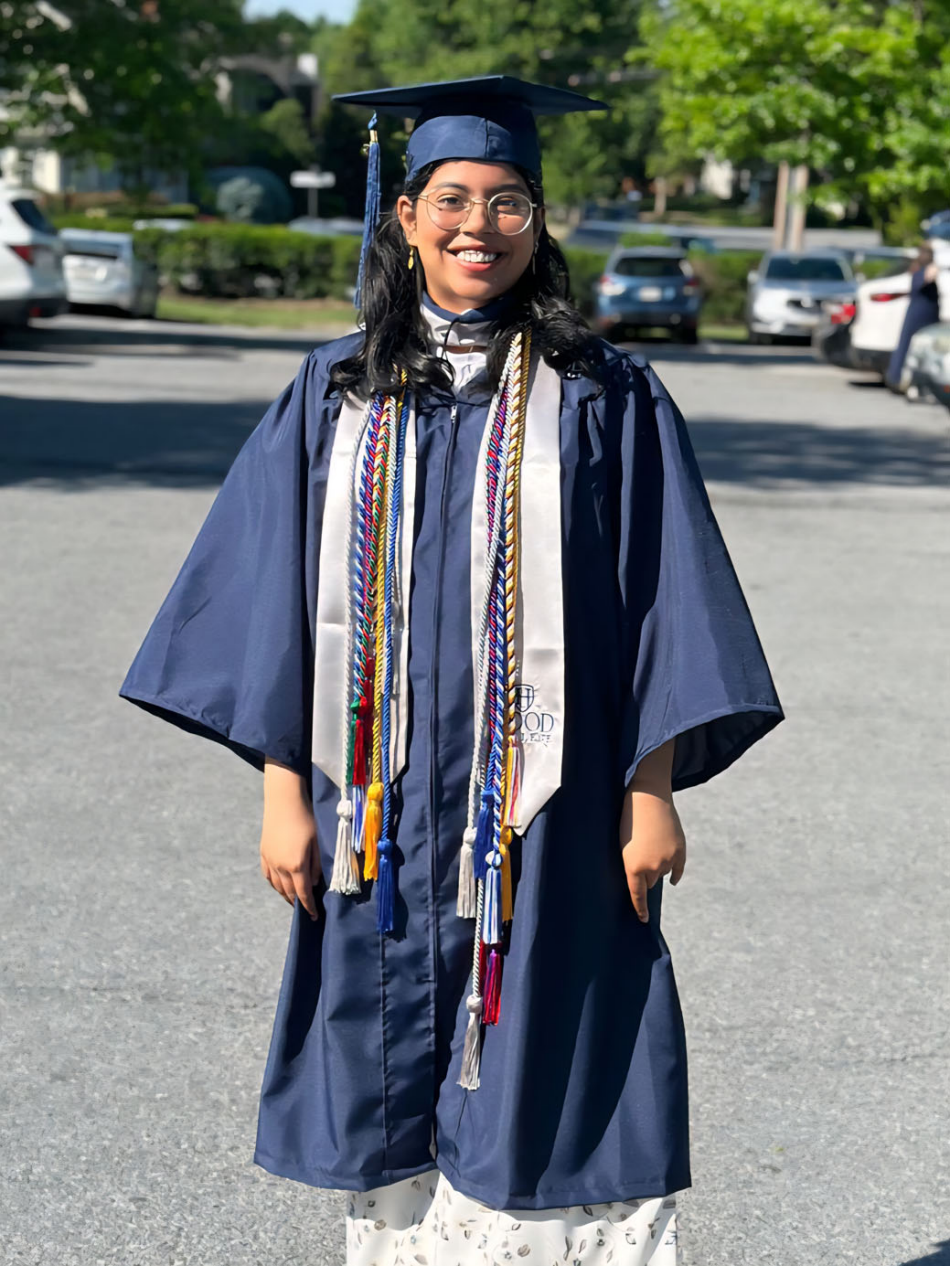


**Khatija Nishat**

**Describe your scientific journey and your current research focus**

My scientific journey began with a broad interest in biology, which narrowed toward cell biology and human disease. Early coursework in molecular and developmental biology sparked my curiosity about how cellular structures regulate signalling pathways. Hands-on research led me to study the primary cilium, an organelle critical for development and signalling. Currently, I focus on intracellular transport proteins, specifically IFT139 (Thm1), and their role in ciliogenesis and signalling. Using wild-type, knockout, and patient-derived mutant rescue cell models, I examined how disruptions in retrograde ciliary transport affect cell proliferation and developmental pathways. This research shed light on ciliary biology and the mechanisms underlying ciliopathies.

**How would you explain the main finding of your paper?**

We found that a tiny cellular structure called the primary cilium, which helps cells ‘talk’ to each other during development, depends on a protein called IFT139 to work properly. When this protein is missing or mutated (as in certain genetic diseases), signals inside the cilium get disrupted, and cells don't grow or respond the way they should. This helps explain how defects in IFT139 can lead to human developmental disorders known as ciliopathies.

**Figure:**
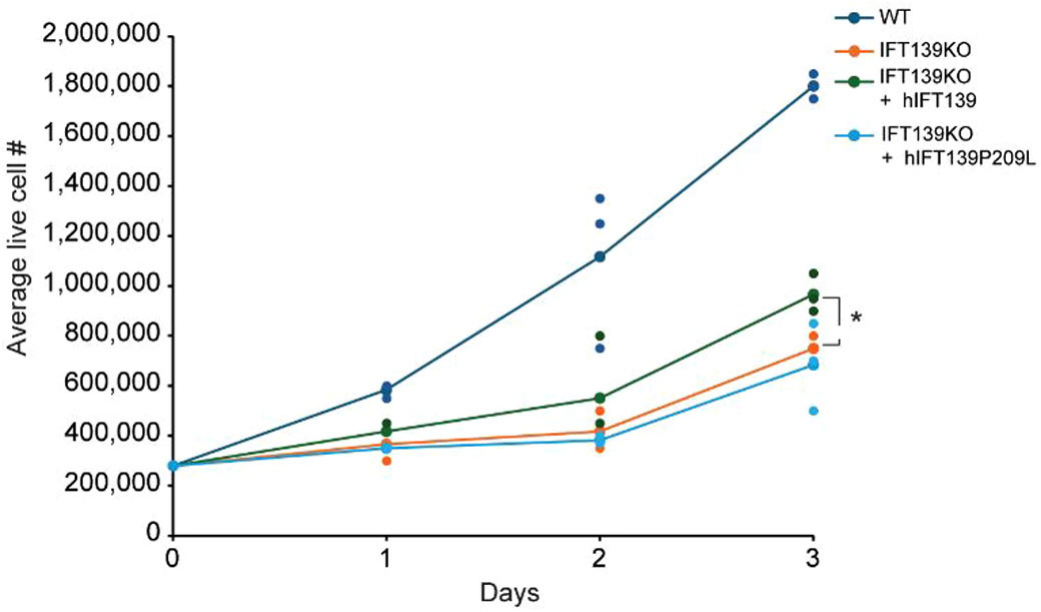
Loss of IFT139 reduces cell proliferation, rescued by wild type but not P209L IFT139.

**Which part of this research project was the most rewarding?**

The most rewarding part of this research was watching the project develop from initial troubleshooting to meaningful results. Early on, it took patience to establish reliable assays for cilia structure and proliferation, but once those experiments started working, the data revealed a clear pattern: the patient-derived P209L mutation impaired cell growth and signalling much like a complete loss of IFT139. That moment was especially exciting, because it connected our lab work directly to the biology of human disease. Being able to see how small changes at the cellular level can help explain complex developmental disorders was both motivating and fulfilling, and it made me feel like our research was contributing to the larger scientific effort to understand ciliopathies.

**What piece of advice would you give to the next generation of researchers?**

Stay curious, don't be afraid to fail, and celebrate the small wins. Ask questions, seek guidance, and remember that the journey of discovery is just as important as the results.

**What's next for you?**

Next, I'm looking forward to gaining more hands-on research experience, exploring new questions, and expanding my skills in whatever area of biology I'm working in. I'm excited to take on projects that challenge me, collaborate with others, and contribute to meaningful discoveries. Ultimately, I aim to continue learning and grow toward graduate studies, where I can further explore questions that connect science to real-world problems.
